# Organelle Membrane Extensions in Mammalian Cells

**DOI:** 10.3390/biology12050664

**Published:** 2023-04-27

**Authors:** Ruth E. Carmichael, David M. Richards, H. Dariush Fahimi, Michael Schrader

**Affiliations:** 1 Department of Biosciences, Faculty of Health and Life Sciences, University of Exeter, Exeter EX4 4QD, UK; 2 Living Systems Institute, University of Exeter, Exeter EX4 4QD, UK; 3 Department of Physics and Astronomy, University of Exeter, Exeter EX4 4QL, UK; 4 Institute for Anatomy and Cell Biology, University of Heidelberg, 69120 Heidelberg, Germany

**Keywords:** organelles, peroxisomes, mitochondria, membrane dynamics, membrane protrusion, nanotubule, organelle interaction

## Abstract

**Simple Summary:**

Within cells, there are numerous compartments called ‘organelles’ that perform a range of specialised functions required to support life. Organelles are constantly adapting to their environment, changing shape and cooperating with each other depending on the cellular needs, which is essential for cell health as defects in these processes lead to human diseases. One example of organelle dynamic behaviour is the formation of thin tubules that extend and retract from the membranes that delimit the organelles. With a focus on two organelles (peroxisomes and mitochondria) that have roles in cell metabolism and protection, we examine how and why these membrane extensions form, and what their function is within the cell. This includes forming new organelles or organelle networks; increasing the organelle surface area to maximise uptake of molecules; mediating communication between different organelles. We propose that these membrane extensions allow organelles to ‘reach out’ and explore their surroundings more efficiently. Together, this review highlights the importance of organelle dynamics, and specifically membrane extension, in maintaining healthy cell function, as well as exploring the questions remaining to be answered to further our understanding of this essential aspect of cell biology.

**Abstract:**

Organelles within eukaryotic cells are not isolated static compartments, instead being morphologically diverse and highly dynamic in order to respond to cellular needs and carry out their diverse and cooperative functions. One phenomenon exemplifying this plasticity, and increasingly gaining attention, is the extension and retraction of thin tubules from organelle membranes. While these protrusions have been observed in morphological studies for decades, their formation, properties and functions are only beginning to be understood. In this review, we provide an overview of what is known and still to be discovered about organelle membrane protrusions in mammalian cells, focusing on the best-characterised examples of these membrane extensions arising from peroxisomes (ubiquitous organelles involved in lipid metabolism and reactive oxygen species homeostasis) and mitochondria. We summarise the current knowledge on the diversity of peroxisomal/mitochondrial membrane extensions, as well as the molecular mechanisms by which they extend and retract, necessitating dynamic membrane remodelling, pulling forces and lipid flow. We also propose broad cellular functions for these membrane extensions in inter-organelle communication, organelle biogenesis, metabolism and protection, and finally present a mathematical model that suggests that extending protrusions is the most efficient way for an organelle to explore its surroundings.

## 1. Introduction

**Figure 1:**
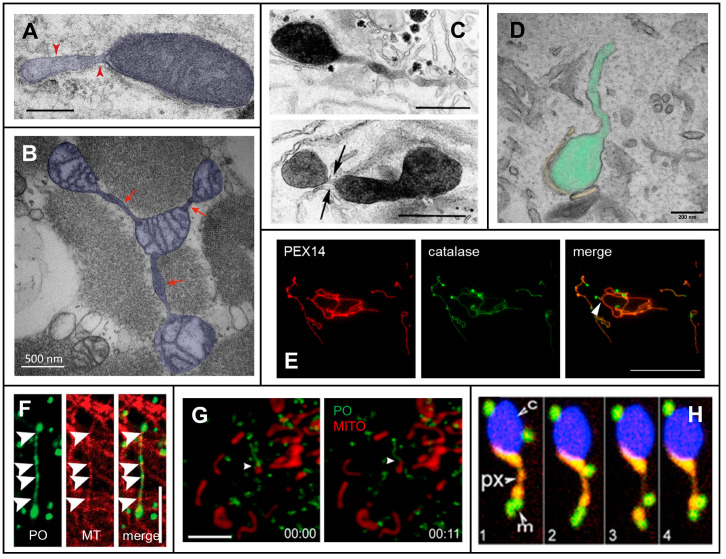
Examples of peroxisomal and mitochondrial membrane extensions (**A**) Electron micrograph of a mitochondrion (blue) in a rat hippocampal neuron displaying a tubulovesicular protrusion (red arrowheads). Bar = 200 nm. Image taken from [17]. (**B**) Electron micrograph of mitochondria (blue) in human skeletal muscle connected by membrane extensions (red arrows), generating a network. Image taken from [16]. (**C**) Electron micrograph showing peroxisomes (darkly stained from the reaction of catalase with 3,3′ diaminobenzidine tetrahydrochloride [DAB]) in regenerating rat liver). The upper panel shows a protrusion emanating from a peroxisome body; the lower panel shows constriction (black arrows) of a tubule prior to fission. Bars = 500 nm. Image taken from [2] with permission from Rockefeller University Press. ©1987 Yamamoto & Fahimi. Originally published in J. Cell. Biol. https://doi.org/10.1083/jcb.105.2.713. (**D**) Electron micrograph of a peroxisome (green) in an MFF-deficient (dMFF) skin fibroblast, where a block in peroxisome fission leads to highly elongated membrane extensions arising from the spherical peroxisome body. Note the peroxisome body is closely associated with the ER (yellow), presumably for membrane lipid transfer to support elongation. Bar = 200 nm. (**E**) Immunofluorescence showing hyper-elongated peroxisomal membrane extensions in a dMFF cell. Peroxisomes were stained with antibodies against PEX14 (peroxisomal membrane marker, red) and catalase (peroxisomal matrix marker, green). The arrowhead indicates a potential tubule branch point. Bar = 20 μm. Image adapted from [18]. (**F**) Immunofluorescence of a peroxisomal protrusion in a PEX5-deficient fibroblast, induced by overexpression of a peroxisomal-targeted version of the motor protein MIRO1 (green). The protrusion runs along microtubule tracks, stained with anti-tubulin (red, indicated by arrowheads). Bar = 5 µm. Image taken from [12]. (**G**) Stills from live-cell imaging of a COS-7 cell expressing the peroxisomal membrane-shaping protein PEX11β-EGFP and stained with Mitotracker Red. A protrusion from a peroxisome (PO, green) can be seen to come into close contact with a mitochondrion (MITO, red). Bar = 5 µm. Image taken from [19]. (**H**) Stills from live-cell imaging of a cotyledon cell from an Arabidopsis mutant exhibiting a high frequency of peroxules, expressing YFP-PTS1 (peroxisomal matrix marker) and mito-GFP (mitochondrial marker). A peroxisome (px, yellow/orange), associated with a chloroplast (c, blue), extends a protrusion that contacts a mitochondrion (m, green). Image taken from [20].

The view of membrane-bound organelles in eukaryotic cells as individual, static entities is outdated. The development of organelle-specific fluorescent markers in combination with advanced live cell microscopy approaches has allowed unprecedented insights into subcellular organelle dynamics, including movement, tubulation, fusion and division. An interesting phenomenon, which now regains attention, is the ability of several organelles, including plastids, peroxisomes and mitochondria, to extend and retract thin membrane tubules (also referred to as protuberances, extensions, or protrusions) of approx. 80–200 nm in diameter and up to 30 µm or more in length. Although such organelle extensions were initially reported in early morphological studies (e.g., [1,2]), their dynamic nature first became apparent in plant cells, where they were named stromules [3,4], peroxules [5,6,7] and matrixules [7,8], respectively (reviewed in [9]). Other organelles in plant cells, e.g., the vacuoles or nuclei, have also been reported to extend tubules but are not well studied [10,11]. There is now evidence that similar dynamic structures exist in mammalian cells, which have been reported for peroxisomes [12] (peroxisomal membrane protrusions; reviewed in [13]) and mitochondria [14,15] (e.g., mitochondrial dynamic tubulation, mitochondrial nanotunnels, nanotubes; reviewed in [16]) (Figure 1). The formation of transient and dynamic tubules, therefore, appears to be a common subcellular phenomenon. It should be noted that the thin, dynamic tubules observed are morphologically different from regular tubular organelles, which are thicker and more static.

Membrane protrusions have gained attention in modern cell biology as they contribute to the communication and connection of organisms, cells and organelles. They include bacterial tubule-like structures, which allow bacteria to exchange cellular molecules (e.g., proteins) with each other [21], as well as cell-to-cell membrane protrusions such as cytonemes and tunnelling nanotubes, which connect individual mammalian cells to enable the transfer of signals or even organelles [22,23]. The widespread appearance of membrane protrusions in biological systems underlines their significance. We summarise and discuss here recent findings about dynamic tubule formation by subcellular organelles in mammalian cells, focussing on mitochondria and particularly peroxisomes. Both are oxidative organelles that closely cooperate in cellular redox balance and lipid metabolism, and even share proteins of their division machinery [24]. For simplicity, and to underline similarities between them, we will refer to the thin membrane tubules as organelle membrane extensions or protrusions in the following.

## 2. Mechanisms of Membrane Protrusion Formation

### 2.1. Dynamic Tubulation of Mitochondria

Membrane extensions depend on cytoskeletal tracks and motor proteins to exert pulling forces on an immobilized organelle. Contrary to plant cells, where the actin cytoskeleton and myosin motors play a role [9,11], in mammalian cells microtubules provide the major tracks along which membrane protrusions form, driven by microtubule-dependent motor proteins such as dynein and kinesin. Live-cell imaging studies revealed that mitochondrial protrusions extend and retract rapidly from mitochondria in a kinesin (KIF5B)- and microtubule-dependent manner [25]. Mitochondrial protrusions were found to align with microtubules, and microtubule depolymerization or loss of KIF5B prevented their formation [14,25,26]. Mitochondrial extensions were also observed in vitro on isolated mitochondria in the presence of polymerized microtubules, KIF5B and ATP [25].

Mitochondrial membrane extensions have been identified in tissues such as the heart [26,27,28], skeletal muscle [29,30,31,32] and brain [33]. In cardiomyocytes, similar but distinct structures termed mitochondrial ‘nanotunnels’ are involved in active intermitochondrial sharing of matrix content and membrane components over long distances [27]. These nanotunnels are narrow double-membraned structures (90–210 nm in diameter, up to 30 µm in length), which can contain matrix and cristae in their lumen (Figure 1). It has been suggested that the nanotunnels in cardiomyocytes differ from intermitochondrial connections in skeletal muscle [15]. Exchange events are proposed to involve kissing junctions, where membrane extensions are in close contact with the membrane of another mitochondrion, which could eventually allow the movements of proteins through a transient pore, or fusion events, which allow mixing of matrices. Mitochondrial fusion depends on large GTPases as follows: the mitofusins MFN1 and MFN2 mediate the fusion of the outer mitochondrial membrane, whereas OPA1 is involved in the fusion of the inner mitochondrial membrane [34].

Mitochondrial extensions are proposed to form especially when mitochondrial mobility is extremely limited, e.g., in cardiac muscle cells that are densely packed with myofibrils [16]. As this restricts the opportunities for mitochondria to be transported to encounter potential fusion partners, extension formation overcomes this problem and allows molecular exchange. In line with this, the formation of mitochondrial extensions was promoted by the inhibition of mitochondrial motility [14].

Different from fusion and division, which regulate mitochondrial connectivity in the majority of the cell, dynamic membrane extensions have been demonstrated to be critical for the formation of the mitochondrial network in the peripheral zones of mammalian cells. Here, a mitochondrial network is generated by repeatedly pulling tubules out of existing mitochondria and connecting them by fusion, forming a membrane bridge that quickly thickens and becomes part of the mitochondrial network [25]. Stable connections between mitochondria can be formed in this manner; indeed, matrix-located green fluorescent protein (GFP) could be transferred from one mitochondrion to another via the transient dynamic tubular connection within seconds, indicating tubulation and fusion of both inner and outer mitochondrial membranes as a mechanism to generate a contiguous matrix between two previously separate mitochondria.

Recently, mitochondrial dynamic tubulation, which predominantly occurs at ER-mitochondria contact sites, has been reported. These mitochondrial extensions play a role in the active transport and proper distribution of mitochondrial DNA (nucleoids) within the mitochondrial network [35], contrasting the long-believed viewpoint that nucleoids are mainly segregated and allocated by constrained diffusive motion within the inner mitochondrial membrane. This active process depends on KIF5B-driven tubulation and involves the mitochondrial inner membrane protein complex MICOS, which links nucleoids to MIRO1, a tail-anchored membrane adaptor for motor proteins/KIF5B at the outer mitochondrial membrane.

Moreover, mitochondrial membrane protrusions have been reported to be involved in the biogenesis of mitochondria-derived vesicles (MDVs) [17,36]. MDVs contribute to mitochondrial quality control and facilitate the delivery of functionally impaired mitochondrial membrane proteins to lysosomes for degradation. A role for MDVs in the delivery of proteins to peroxisomes has also been suggested [37]. MDV formation appears to be initiated by MIRO1/2-dependent microtubule-mediated pulling of thin membrane protrusions out of mitochondria. Then, the fission GTPase DRP1 is recruited by the mitochondrial adaptor proteins MID49, MID51 and MFF and mediates vesicle scission at the tip of the tubule. It is possible that mitochondrial protrusions in plant cells also contribute to MDV formation [38].

Furthermore, stress conditions can induce mitochondrial extensions, e.g., dysregulation of Ca^2+^ homeostasis through ryanodine receptor dysfunction in cardiomyocytes [26], manganese exposure or complex III inhibition by Antimycin A in neurons [17,33]. Mitochondrial extensions can also be the result of incomplete mitochondrial fission or impairment of the mitochondrial division machinery [39]. Similarly, in plant cells, mitochondrial extensions have also been linked to mitochondrial fission and are more frequent when mitochondrial division is impaired [8,9].

In mammalian cells, mitochondrial membrane extensions are so far mainly linked to the interaction and cooperation of mitochondria with other mitochondria. However, in plant cells, membrane extensions of plastids (stromules) are proposed to transport metabolites and proteins between cellular compartments [9]. Furthermore, peroxisomal membrane extensions in plants and mammalian cells have been observed to dynamically interact with mitochondria [19,20] (Figure 1). Membrane extensions (e.g., of plastids) are as well postulated to increase organelle surface area to facilitate the exchange efficiency between the cytosol and the organelle.

### 2.2. Peroxisomal Membrane Protrusions

**Figure 2:**
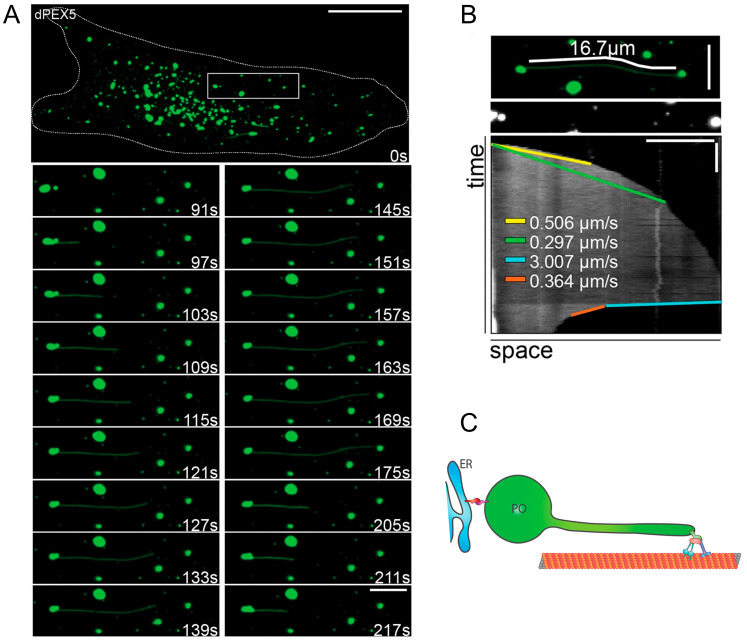
Dynamics of peroxisomal membrane protrusions. Time-lapse of MIRO1-expressing PEX5-deficient fibroblasts labelled with a peroxisomal membrane marker (green). (**A**) Membrane protrusions form from enlarged (non-functional) peroxisomal membranes (ghosts), elongate and retract. Bars = 20 μm (overview), 5 μm (magnification). (**B**) Kymograph of peroxisome elongation observed in (**A**). Note the high velocity during tubule retraction. Bars = 20 s (vertical), 5 μm (horizontal). (**C**) Schematic of protrusion forming from an ER-tethered peroxisome (PO) due to MIRO1-mediated motor forces acting along the microtubule cytoskeleton. Images taken from [12].

Similarly to mitochondrial tubules, peroxisomal membrane protrusions are aligned along microtubule tracks, and microtubule depolymerisation alters their morphology [12,18]. While proteins that mediate the formation as well as the extension–retraction behaviour of organelle/peroxisome extensions have not been identified in plants [9], there is some insight from mammalian cells. The Rho GTPase MIRO, which is a tail-anchored membrane protein at mitochondria (see Section 2.1) and peroxisomes, can interact with kinesin and dynein motor proteins and drive membrane extensions. Overexpression of MIRO1 can promote the formation of peroxisomal membrane extensions, which are observed to extend and retract along microtubules [12] (Figure 2). Interestingly, long peroxisomal extensions (>20 µm) can also bend to follow another microtubule at crossover points and even be branched (unpublished observations) [12]. These observations indicate that docking proteins must exist, which link the membrane tubules to microtubules. A potential candidate is PEX14, a peroxisomal membrane protein with a major function in matrix protein import. PEX14 also interacts with tubulin in vitro, and its N-terminal tubulin binding region has been successfully used as a tool to label microtubules in mammalian cells [40,41]. Furthermore, silencing of PEX14 alters the morphology of membrane extensions, and the frequency of their formation and protrusion length is reduced in PEX14 knockout cells expressing a peroxisomal MIRO1 to promote tubule formation [12]. In addition, PEX14 is preferentially associated with highly elongated peroxisomal membrane extensions in MFF-deficient patient fibroblasts, where peroxisomes are unable to divide [18]. This unequal distribution of PEX14 may serve to stabilise the hyper-elongated peroxisomal extensions by docking them to microtubules. There are, however, conflicting data about the exact membrane topology of PEX14, as the N-terminus appears to be protease-protected and may thus not be accessible to interact with microtubules [42]. It may be possible that different complexes of PEX14 exist (e.g., for matrix protein import or microtubule interaction), which display different conformations. It should also be noted that loss of PEX14 does not inhibit peroxisomal motility in mammalian cells, which would be consistent with a function of a microtubule-docking protein.

#### 2.2.1. Peroxisomal Shaping Proteins

In principle, the pulling action of motors bound to a lipid bilayer is sufficient to generate membrane tubes [43,44]. In line with this, peroxisomal membrane extensions can also form from peroxisomal “ghosts” in peroxisome-deficient cells. Defects in PEX proteins of the peroxisomal import machinery for matrix proteins result in “empty” peroxisomal membranes (ghosts), which are metabolically inactive. Peroxisomes are usually reduced in number and can be enlarged, e.g., in PEX5 deficient cells, which lack a functional import receptor for peroxisomal cargo proteins with a peroxisomal targeting signal 1 (PTS1). Defects in PEX genes can result in severe peroxisome biogenesis disorders such as Zellweger spectrum disorders [45]. As peroxisomes in PEX5-deficient cells are enlarged (≥1 µm vs. 0.2–0.3 µm in control cells), the detection and visualisation of membrane protrusions are more obvious. Overexpression of peroxisomal MIRO1 to exert MIRO1/motor protein pulling forces at the enlarged peroxisomes resulted in the formation of long, dynamic membrane protrusions, which can rapidly extend and retract (Figure 2). This indicates that peroxisomal metabolism is not required for membrane protrusion [12]. Interestingly, shorter membrane protrusions were also observed in PEX5-deficient cells without overexpression of MIRO1. This observation may indicate that membrane extensions may be more frequent than previously expected in mammalian cells and/or may form under certain stress conditions to maintain cellular homeostasis (see Section 3).

The integral peroxisomal membrane-shaping protein PEX11β is a key regulator of peroxisomal membrane dynamics and division [46]. Amphipathic helixes in the N-terminus of PEX11 proteins enable its interaction with membrane lipids to destabilise the outer leaflet and can elongate liposomes in vitro [47]. Together with PEX11β oligomerisation, this is thought to generate the forces required to deform and elongate peroxisomal membranes [48]. Knockdown of PEX11β in PEX5-deficient cells inhibited the formation of membrane protrusions generated by overexpression of peroxisomal MIRO1 [19]. These observations indicate that, besides the cytoskeleton and associated motor proteins, PEX11β is also required for the formation of peroxisomal membrane protrusions in mammalian cells and that peroxisomes do not need to be metabolically active for PEX11β to mediate protrusion formation. The former is in agreement with studies in plants, where *At*PEX11a has been reported to mediate the formation of peroxisomal membrane extensions in response to ROS [49]. Besides protrusion formation, PEX11β also promotes the growth/elongation of peroxisomal membranes, which is a pre-requisite for the division and multiplication of peroxisomes. In addition to its membrane-deforming activity, PEX11β interacts with components of the division machinery, such as the membrane adaptors FIS1 and MFF, which recruit the fission GTPase DRP1 to peroxisomes (and mitochondria) (reviewed in [46]). PEX11β also stimulates the GTPase activity of DRP1, potentially by promoting DRP1 oligomerisation at constriction sites [50]. The formation of peroxisomes by growth and division out of pre-existing organelles follows a multi-step process, which involves membrane deformation and elongation, constriction and final division, with PEX11β playing roles in all steps. Loss of DRP1 or MFF function, as observed in patients with defects in peroxisomal (and mitochondrial) dynamics, results in highly elongated peroxisomes (and mitochondria) (reviewed in [51]). In particular, the loss of MFF, a major membrane adaptor for DRP1, causes the formation of hyper-elongated peroxisomal membrane protrusions, which emanate from a spherical peroxisome (Figure 1). These membrane protrusions are highly dynamic; they can rapidly extend and retract, branch, and show transient dilations (unpublished observations), similar to what was observed in plant cells [9]. The peroxisomal membrane extensions in MFF-deficient cells are thinner than the tubular peroxisomes, which undergo fission and appear to represent a pre-mature membrane compartment, which is not yet import-competent for peroxisomal matrix proteins [18]. In this respect, it has also been suggested that the membrane protrusions in plant cells represent intermediate forms between spherical and tubular structures [9]. Long peroxisomal membrane protrusions have also been observed in yeast cells lacking the fission GTPases Dnm1 or Vps1 [52,53]. These protrusions depend on Pex11 and use the actin cytoskeleton and myosin motors, which are recruited to peroxisomes via the membrane adaptor Inp2, a crucial protein for organelle inheritance in budding yeast cells (reviewed in [54]).

Supporting the idea that peroxisome tubules are an intermediate morphology preceding division into spherical organelles, we recently revealed that overexpression of PEX11β in MFF-deficient patient cells promotes the division of the hyper-elongated peroxisome tubules into spherical organelles [55]. This observation was surprising, as (i) MFF was suggested to be the major recruitment factor for DRP1 at peroxisomes [56] and (ii) PEX11β has no intrinsic division activity [57]. We then demonstrated that the PEX11β-mediated division of these peroxisomes depends on DRP1 and FIS1. The latter is also a membrane adaptor for DRP1 but needs to cooperate with PEX11β to mediate peroxisome division. Whereas the N-terminal region of PEX11β is important for membrane lipid interaction and oligomerisation, the short cytoplasmic C-terminus is essential to promote peroxisome division in MFF-deficient cells [55]. The C-terminal region may be required to form a functional division complex with FIS1 and DRP1. These findings also point to the existence of the following two independent division pathways of peroxisomes in mammalian cells: one depending on MFF, the other on PEX11β-FIS1. As MFF is only found in metazoa, the PEX11β-FIS1-driven pathway may be the evolutionary older one. As MFF and FIS1 are shared by peroxisomes and mitochondria, whereas PEX11β is peroxisomal, this may allow independent and coordinated regulation of peroxisomal and mitochondrial division.

#### 2.2.2. Peroxisome-ER Interaction, Tethers and Lipid Flow

Initial calculations of the surface area of spherical peroxisomes and the membrane protrusions emanating from those spherical organelles indicated that the surface area of the membrane protrusions was several-fold larger than the surface area of the globular mother peroxisome [12,18]. Thus, the latter is unlikely to contain sufficient amounts of lipids to enable the formation of such long protrusions. An explanation came through the discovery of tether proteins, which link peroxisomes to the ER [58,59]. We revealed that ACBD5 (acyl-CoA binding domain protein 5), a tail-anchored peroxisomal membrane protein, interacts with ER-resident VAP proteins to tether both organelles and form membrane contact sites [58]. The interaction is mediated by an FFAT motif in ACBD5, which interacts with the MSP domain of VAP proteins and is regulated by the phosphorylation of ACBD5 [60]. Interestingly, overexpression of ACBD5 resulted in peroxisome elongation, whereas overexpression of a phospho-mutant, which blocked peroxisome-ER interaction, did not elongate peroxisomes. Furthermore, loss of ACBD5 in MFF-deficient patient fibroblasts caused a reduction in the length of the peroxisomal protrusions. Interestingly, ACBD5 localises to the globular peroxisomes, which give rise to the membrane protrusions. Electron microscopy revealed that the globular peroxisomes were in close contact with the ER [61]. These observations indicate that membrane lipids for peroxisomal membrane expansion are delivered from the ER to peroxisomes via ACBD5-VAP-mediated membrane contact sites. As an artificial tether could restore peroxisome elongation after the loss of ACBD5 in MFF-deficient fibroblasts [58], it is unlikely that ACBD5 is itself involved in the transport of membrane lipids. Recently, the role of VPS13 proteins in peroxisome biogenesis and lipid transfer has been suggested [62,63]. VPS13 proteins are large, bridging proteins which can form a channel between organelles to allow a bulk flow of lipids (reviewed in [64,65]). Such a mechanism would be consistent with the highly dynamic nature of the peroxisomal membrane protrusions [12]. Mathematical modelling of peroxisome dynamics based on experimental data and simulations supports the observations in MFF-deficient cells. A simple model has been developed, which is based on ER to peroxisome lipid flow rate, elongation and division rates [12,18]. A slight reduction in the division rate in the model results in highly elongated peroxisomes, as observed in MFF-deficient cells, where division is blocked. This indicates that a constant flow of lipids from the ER under these conditions leads to peroxisome elongation. Reducing the lipid flow rate in the model leads to shorter peroxisomes and mimics the experimental observations after the loss of ACBD5, which results in shorter peroxisomes in MFF-deficient cells. 

It should be noted that the peroxisome-ER membrane contacts also fulfil metabolic functions, e.g., in cooperative ether lipid synthesis between peroxisomes and the ER, as well as in fatty acid metabolism. Patients with a defect in ACBD5 have been identified and suffer from leukodystrophy and retinopathy [51,66,67]. These patients show an accumulation of very-long-chain fatty acids (VLCFA), which can only be degraded in peroxisomes via the peroxisomal β-oxidation pathway. ACBD5 has a preference for VLCFA and is hypothesised to capture them via its acyl-CoA binding domain, and to route them to the peroxisomal ABC transporter ABCD1 for uptake into peroxisomes and subsequent β-oxidation. In addition, the ACBD5-VAP-mediated peroxisome ER contacts are important for organelle positioning and mobility. Loss of ACBD5 increases the mobility and net displacement of peroxisomes [58], indicating that tethering to the ER reduces peroxisome mobility. In fibroblasts, 70–80% of the peroxisome are in contact with the ER, explaining the moderate number of mobility events observed in live cell imaging [68]. Interestingly, the expression of a peroxisomal MIRO1 in fibroblasts to exert pulling forces at peroxisomes resulted in increased peroxisome numbers, likely by promoting division and pulling peroxisomes apart [12]. This is different in other cell types (e.g., COS-7 cells), where MIRO1/motor protein-mediated pulling relocates peroxisomes to the cell periphery. Interestingly, peroxisome-ER contacts are less frequent in COS-7 cells, presumably facilitating this relocation rather than membrane elongation and division [12]. These observations indicate that in order for membrane expansions to form through motor-mediated forces, peroxisomes need to be tethered/immobilized to other structures, e.g., the ER or the cytoskeleton. Curiously, the depolymerisation of microtubules (but not their stabilisation) has been shown to promote peroxisome elongation, which is followed by division and multiplication [69,70]. It is likely that PEX11β can elongate and promote the division of peroxisomes in the absence of microtubules; however, these processes may be facilitated by microtubules, and their loss may reduce the division rate, thus resulting in elongation.

## 3. Possible Functions of Organelle Membrane Extensions

In the following section we are considering the broader functions of membrane extensions shared between peroxisomes and mitochondria.

### 3.1. A Role for Membrane Protrusions in Organelle Biogenesis and Dynamics

**Figure 3:**
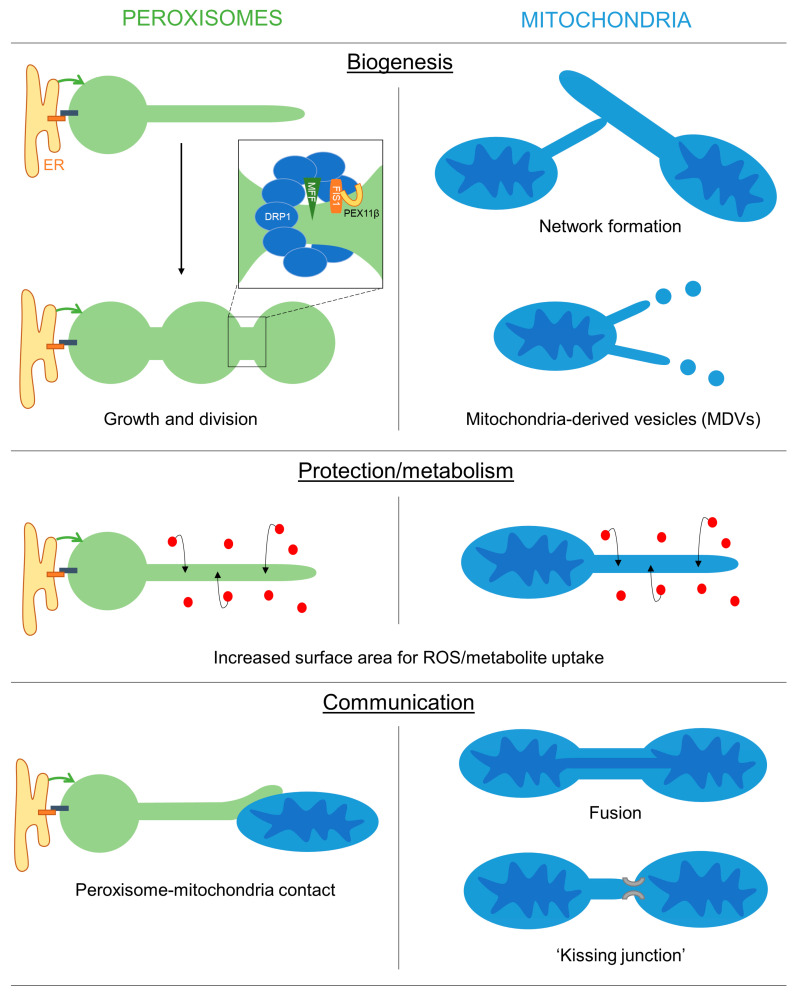
Overview of the possible functions of peroxisomal and mitochondrial membrane extensions. Schematic showing the potential roles of peroxisomal/mitochondrial membranes extensions in mediating organelle biogenesis, protection/metabolism and inter-organelle communication.

As outlined above (see Section 2.2), peroxisomal membrane extensions are linked to the growth and division process of peroxisomes for multiplication/proliferation (Figure 3). PEX11β-mediated membrane deformation and protrusion formation can result in the generation of tubular peroxisomes, which subsequently constrict and divide involving MFF, FIS1 and DRP1. The initial thin membrane protrusions appear to be an intermediate between the spherical and tubular states, as they accumulate in cells with a defect in peroxisome division (e.g., in MFF deficiency). This is also observed in plant cells [8,9] and in yeast. In yeast, loss of the fission GTPases Dnm1 and/or Vps1 results in enlarged peroxisomes, which can form long protrusions [52,53]. The latter emanates towards the budding site in an attempt to deliver and inherit peroxisomes to the daughter cell, which is occasionally successful in the mutants. Protrusion formation under those conditions depends on Pex11 as Dnm1/Pex11 mutants are unable to generate peroxisomal protrusions [53]. 

One function of membrane extensions is therefore linked to peroxisome formation. The latter is influenced by environmental conditions (e.g., the presence of peroxisomal substrates such as VLCFA) involving cellular signalling processes (e.g., via PPARs). However, the signalling pathways that regulate peroxisome multiplication/proliferation in humans are not well understood [71]. Our modelling approach revealed that the presence and frequency of peroxisomal protrusions and tubules are largely dependent on the lipid flow, elongation and division rates in different cell types (see above) [12,18,46]. For example, in human skin fibroblasts, peroxisomes are mainly spherical but can massively hyper-elongate when division is blocked. This indicates that the lipid flow rate, elongation and division rates must be high in fibroblasts and protrusions, or elongated peroxisomes are rarely captured due to the fast dynamics/turnover. 

Similarly, membrane extensions of mitochondria contribute to mitochondrial dynamics and the formation of the mitochondrial network in the peripheral zones of mammalian cells (see Section 1.). The tubular membrane bridges that form, quickly thicken and become part of the mitochondrial network, indicating they represent an intermediate during network formation. Furthermore, mitochondrial extensions have been linked to incomplete DRP1-mediated mitochondrial fission [39]. Finally, mitochondrial protrusions contribute to the biogenesis of MDVs [36].

### 3.2. A Protective Role for Organelle Membrane Protrusions

In plant cells, the formation of peroxisomal membrane protrusions (peroxules) is promoted by high levels of reactive oxygen species (ROS), e.g., during exposure to high-intensity light [20,49,72,73,74]. Remarkably, thin peroxules form within seconds following exposure to ROS and are suggested to contribute to a rapid uptake and neutralization of these damaging radicals [20,72,73,75]. This may be achieved by the increased surface area to volume ratio [76,77], which could facilitate the ROS exchange efficiency between the cytosol and the organelle (Figure 3). Persistent ROS stress results in a complete tubulation of peroxisomes, which ultimately divide and multiply to increase peroxisome numbers in the plant cell [72]. Tubulation and division of peroxisomes in mammalian cells after exposure to H_2_O_2_ or UV irradiation have also been observed [78]. However, the generation of oxidative stress in mammalian peroxisomes, mitochondria or the cytosol, e.g., by expressing organelle-targeted KillerRed, did not result in morphological alterations of peroxisomes [79]. In MFF-deficient fibroblasts with hyper-elongated peroxisomes and mitochondria, no changes in the oxidation state were observed in the cytosol and mitochondria using an H_2_O_2_-responsive variant of roGFP2, whereas peroxisomes showed reduced levels of H_2_O_2_ when compared to control cells [18].

Similarly, stress conditions have been reported to promote the formation of mitochondrial extensions, e.g., through Ca^2+^ dysregulation, manganese exposure or complex III inhibition (see Section 1.). The protective effect of those extensions may primarily lie in the increased interconnectivity of mitochondria. In addition, stress conditions that impair the function of mitochondrial membrane proteins/complexes promote membrane extension and subsequent MDV formation to facilitate lysosomal delivery and degradation of those mitochondrial proteins [36].

### 3.3. Organelle Protrusions, Communication and Metabolic Exchange

Interestingly, in plant cells, peroxules also interact with mitochondria, presumably to prevent damage to those ROS-distressed organelles [20,74]. An interaction between peroxisomal protrusions and mitochondria was also observed in mammalian cells after overexpression of PEX11β, which promotes membrane expansion [19] (Figure 1). Interactions of elongated peroxisomes with mitochondria were more frequent than those of spherical organelles, but both interactions were long-lasting. Interestingly, in a large-scale mapping approach, PEX11β was found to be co-regulated with proteins of the mitochondrial ATP synthase complex, suggesting coordination of peroxisomal and mitochondrial functions. MIRO1, a membrane adaptor for the microtubule-dependent motors kinesin and dynein, was also co-regulated with PEX11β. In yeast, potential peroxisome-mitochondria tether proteins (e.g., Pex11-Mdm34, Pex34, Fzo1) have been identified [80,81], and contacts between both organelles appear to facilitate the transfer of acetyl-CoA from peroxisomes to mitochondria for efficient fatty acid degradation and energy generation [81].

An additional function of peroxisomal membrane protrusions may therefore be to facilitate organelle interaction and communication (Figure 3). Peroxisomes and mitochondria are both oxidative organelles, which contribute to cellular redox balance, but also cooperate in the β-oxidation of fatty acids in mammalian cells. This requires the transfer of chain-shortened fatty acids from peroxisomes to mitochondria and the exchange of cofactors. Peroxisomal protrusions, which interact with mitochondria, may therefore facilitate metabolic exchange as well as contribute to redox homeostasis. Similarly, in yeast, peroxisomes and lipid droplets can interact through peroxisomal extensions (called pexopodia) that extend into lipid droplets to facilitate the diffusion of fatty acids for β-oxidation in peroxisomes [82,83]. In plant seeds, peroxisomal extensions are also reported to deliver the Arabidopsis SDP1 lipase to oil bodies for triacylglycerol degradation and fatty acid mobilization [84].

As in mammalian cells, 70–80% of the peroxisomes can be engaged in close contact with the ER, which often wraps around peroxisomes, this causes a potential problem in terms of reconciling this immobilization with the need for direct interaction and simultaneous cooperation with mitochondria. The peroxisome-ER contacts are important for ether lipid synthesis and lipid transfer, whereas their interaction with mitochondria is important for fatty acid- and co-factor exchange [85]. Peroxisome membrane expansion may overcome this problem and allow the peroxisomes to stay tethered to the ER while also interacting with mitochondria (or other organelles). The membrane protrusions may therefore represent an alternative, more dynamic form of organelle contact site, which supports simultaneous interaction and communication with a third organelle without changing position.

**Figure 4:**
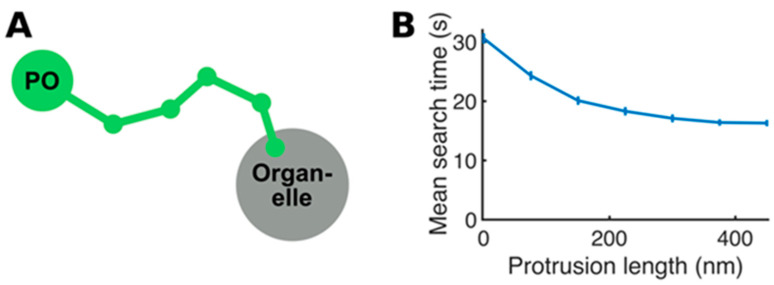
Mathematical model of organelle searching. (**A**) Representation of the peroxisome (PO) in the model, with a spherical body and an optional piece-wise extension (consisting of vertices connected by straight segments) that represent the protrusion. (**B**) Mean search time as a function of the protrusion length, showing that a protrusion can significantly decrease the organelle search time. Note that for simplicity, the cell was assumed to be a sphere of radius 1 μm. Error bars show the standard error of the mean. See Appendix A for a detailed explanation of the model generation.

To test whether protrusion formation might enable peroxisomes to find other organelles quicker than for non-extended peroxisomes, we designed and simulated a simple mathematical model. The model was deliberately kept simple (Figure 4 and Appendix A) to allow the underlying benefit of tubular-versus-spherical searching to be studied without being obscured by overly complicated details such as the precise components and biophysics of the peroxisomal membrane. This model Indeed showed that protrusions result in quicker organelle searching, with the more dynamic extension able to search more efficiently through the cytoplasm. Further, the longer the protrusion, the shorter the average search time (Figure 4). Interestingly, however, the benefit of longer protrusions gradually decreases as follows: for sufficiently long protrusions, further increasing the protrusion length leads to little additional decrease in search time. The generation of a tentacle-like protrusion may thus allow the exploration of a given space more quickly and efficiently. This could then be followed by a more directed microtubule-based membrane extension.

Mitochondrial extensions can connect individual mitochondria, either by fusion or by kissing junctions, which allow the exchange of proteins and metabolites (see Section 1). Although peroxisomes share components of the division machinery with mitochondria, they do not share fusion proteins such as MFN1/2 or OPA1 and have not been reported to fuse or exchange fluorescent matrix proteins through a mechanism similar to mitochondrial fusion [86,87]. However, they do show “kiss and run” behaviour and self-interaction [86] and can form reticular-like structures [88]. Nevertheless, it is likely that peroxisome extensions, which contact other organelles, do not result in fusion, but instead form kissing junctions or contact sites. If specific tether proteins are involved in these contacts and if they are identical to the ones already described at mammalian peroxisomes (e.g., ACBD5, ACBD4 involved in peroxisome-ER interaction) is currently unknown. Organelle interactions by membrane extensions may increase the surface area, creating a membrane interface that would facilitate the exchange of metabolites through organelle-specific transport mechanisms [89].

It is also intriguing that membrane extensions are more frequently formed when organelles are immobilized, e.g., by physical constraints, as observed for mitochondria in skeletal and cardiac muscle cells that are densely packed with myofibrils. Tethering of peroxisomes to the ER also results in immobilization and is linked to protrusion formation (see Section 2.2.2). Low numbers of peroxisomes, as observed in PEX5-deficient or MFF-deficient cells, limit the frequency of potential contact events with other organelles. We propose that these conditions also promote protrusion formation to compensate for reduced numbers and maintain the interaction and metabolic cooperation with other subcellular organelles, such as mitochondria and lipid droplets, which all contribute to cellular lipid metabolism. Overall, membrane protrusions appear to accomplish long-range interactions to maintain cellular homeostasis.

## 4. Conclusions

Besides structural differences (e.g., double membrane-bound mitochondrial vs. single membrane-bound peroxisomal protrusions), mechanistic and functional similarities exist between organelle membrane extensions in mammalian cells. Their formation is generally supported by microtubules and associated motor proteins such as kinesin that are recruited to the organelle via the tail-anchored membrane adaptors MIRO1/2 and exert pulling forces at immobilized organelles. The ER also plays a role in protrusion formation, e.g., in the delivery of lipids for membrane expansion as shown for peroxisomes, or in the mechanism of nucleoid distribution through dynamic tubulation of mitochondria at ER-mitochondria contact sites. Similar to observations in plant cells, the ER may also define the paths for organelle membrane extensions.

Membrane extensions appear under certain conditions, such as physical constraints or low organelle numbers, which lead to limitations in organelle contact. In addition, cellular stress conditions can promote membrane extensions. Functionally, they drive an increase in surface area, which can facilitate exchange with the cytosol and subsequent removal of otherwise damaging molecules such as ROS or fatty acids. Membrane extensions also allow the organelles to explore the intracellular environment more efficiently and to engage with other organelles for metabolic cooperation and exchange. This can be achieved by fusion (in the case of mitochondria), kissing junctions or membrane contacts; however, the exact molecular mechanisms for the exchange of material are not well understood. We propose that peroxisomal membrane protrusions are indeed nanotubes, which facilitate the interaction and metabolite exchange with other subcellular organelles. This could be achieved by a more dynamic type of membrane contact site, which may differ from the “classical” tether-based contacts described. Additionally, membrane protrusions contribute to the biogenesis processes of the organelles, e.g., network formation and nucleoid distribution of mitochondria, MDV biogenesis or peroxisome formation/multiplication by membrane growth and division. Those processes may be triggered by certain stress conditions with the purpose to protect the cell and to maintain cellular homeostasis but may also be relevant under physiological conditions. The type, repetitiveness and duration of the stress are likely to influence protrusion formation and the subsequent conversion of these thin intermediates into tubular organelle structures.

Certainly, a challenge is to experimentally capture organelle protrusions in mammalian cells or tissues. Recent developments in microscopy now allow the diffraction barrier of light to be overcome [90,91,92,93,94]. These approaches also enable the visualization of lipids at the nanoscale [95,96,97,98] and will prove advantageous for the further detailed analysis of membrane nanotubes.

Furthermore, their frequency likely depends on the cell type and environmental conditions (see Section 3.2), e.g., the cytosolic distribution of energy-utilizing systems, which impacts mitochondrial morphology [31]. Peroxisomal membrane protrusions may also be more frequent in cell types with lower numbers of peroxisomes or with physical barriers (e.g., extensive peroxisome-ER tethering), which prevent peroxisome-organelle interaction. More work will be necessary to determine the molecular mechanisms that regulate organelle protrusion formation in mammalian cells, including the molecular components involved, the signalling mechanisms triggering dynamic membrane extension, and their specific functions. In addition, the exact mechanisms of contact formation and exchange of metabolites are not well understood and require further investigation. The physiological significance of organelle extensions for the cell and the organism needs to be resolved, as well as their relevance to disease. As organelle protrusions exert protective functions, the ability to modulate them under stress-related disease conditions may offer new therapeutic options for age-related disorders. A better understanding of organelle protrusion formation will also foster our understanding of organelle communication in general, as well as the factors and signals that orchestrate this complex cellular process in human health and disease.

## Data Availability

All datasets generated for this study are included in the article.

## Appendix A

### Details of the Mathematical Model

The model idealises a peroxisome as consisting of a spherical body to which a protrusion is attached (Figure 4). The protrusion consists of N vertices (N = 0 for the case of a non-elongated peroxisome), each initially separated by a straight segment of length ΔL_0_ = 75 nm. We simulated the motion of the peroxisome in the 3D environment of the cell (assumed for simplicity to be sphere of radius 1 μm) as it searches for a fixed organelle (assumed to be a sphere of radius of 0.1 μm). Of course, in reality, the organelle may not be fixed, but, as long as organelle motion is slow compared to that of the peroxisome (or its protrusion), this is unlikely to affect our results much. Peroxisome motion is assumed to be chiefly governed by diffusion. More complicated future models could additionally consider active motion (along, for example, elements of the cytoskeleton), although a purely diffusive mechanism is sufficient for our present purposes. Peroxisome motion consists of two parts: motion of the body and motion of the protrusion. The body is assumed to diffuse as a point particle with diffusion constant D = 0.01 μm^2^/s. On the other hand, for the protrusion, each vertex individually tries to diffuse with a larger diffusion constant of D = 0.1 μm^2^/s. However, since the vertices of the protrusion form a connected object, there are two constraints on their position relative to each other. First, if the current gap between two connected vertices ΔL tries to move further away from ΔL_0_, then this is only accepted with a probability p drawn from a Gaussian with mean ΔL_0_ and standard deviation 0.1 ΔL_0_. Second, if the absolute value of the angle between two adjacent segments of the protrusion tries to increase, then this is only accepted with probability p drawn from a Gaussian with mean 0 and standard deviation 1 radian. We use a time step of Δt = 1 ms and a spatial jump of Δx = 0.02 μm.

## References

[1] Grainger F., James D.W. (1969). Mitochondrial Extensions Associated with Microtubules in Outgrowing Processes from Chick Spinal Cord In Vitro. J. Cell Sci..

[2] Yamamoto K., Fahimi H.D. (1987). Three-Dimensional Reconstruction of a Peroxisomal Reticulum in Regenerating Rat Liver: Evidence of Interconnections between Heterogeneous Segments. J. Cell Biol..

[3] Köhler R.H., Hanson M.R. (2000). Plastid Tubules of Higher Plants Are Tissue-Specific and Developmentally Regulated. J. Cell Sci..

[4] Köhler R.H., Cao J., Zipfel W.R., Webb W.W., Hanson M.R. (1997). Exchange of Protein Molecules through Connections between Higher Plant Plastids. Science.

[5] Jedd G., Chua N.-H. (2002). Visualization of Peroxisomes in Living Plant Cells Reveals Acto-Myosin-Dependent Cytoplasmic Streaming and Peroxisome Budding. Plant Cell Physiol..

[6] Cutler S.R., Ehrhardt D.W., Griffitts J.S., Somerville C.R. (2000). Random GFP::CDNA Fusions Enable Visualization of Subcellular Structures in Cells of Arabidopsis at a High Frequency. Proc. Natl. Acad. Sci. USA.

[7] Scott I., Sparkes I.A., Logan D.C. (2007). The Missing Link: Inter-Organellar Connections in Mitochondria and Peroxisomes?. Trends Plant Sci..

[8] Logan D.C., Scott I., Tobin A.K. (2004). ADL2a, like ADL2b, Is Involved in the Control of Higher Plant Mitochondrial Morphology. J. Exp. Bot..

[9] Mathur J. (2021). Organelle Extensions in Plant Cells. Plant Physiol..

[10] Wiltshire E.J., Collings D.A. (2009). New Dynamics in an Old Friend: Dynamic Tubular Vacuoles Radiate through the Cortical Cytoplasm of Red Onion Epidermal Cells. Plant Cell Physiol..

[11] Mathur J., Mammone A., Barton K.A. (2012). Organelle Extensions in Plant Cells. J. Integr. Plant Biol..

[12] Castro I.G., Richards D.M., Metz J., Costello J.L., Passmore J.B., Schrader T.A., Gouveia A., Ribeiro D., Schrader M. (2018). A Role for Mitochondrial Rho GTPase 1 (MIRO1) in Motility and Membrane Dynamics of Peroxisomes. Traffic.

[13] Schrader M., Fahimi H.D. (2006). Growth and Division of Peroxisomes. Int Rev. Cytol..

[14] Bowes T., Gupta R.S. (2008). Novel Mitochondrial Extensions Provide Evidence for a Link between Microtubule-Directed Movement and Mitochondrial Fission. Biochem. Biophys. Res. Commun..

[15] Lavorato M., Formenti F., Franzini-Armstrong C. (2020). The Structural Basis for Intermitochondrial Communications Is Fundamentally Different in Cardiac and Skeletal Muscle. Exp. Physiol..

[16] Vincent A.E., Turnbull D.M., Eisner V., Hajnóczky G., Picard M. (2017). Mitochondrial Nanotunnels. Trends Cell Biol..

[17] Yao P.J., Eren E., Petralia R.S., Gu J.W., Wang Y.-X., Kapogiannis D. (2020). Mitochondrial Protrusions in Neuronal Cells. iScience.

[18] Passmore J.B., Carmichael R.E., Schrader T.A., Godinho L.F., Ferdinandusse S., Lismont C., Wang Y., Hacker C., Islinger M., Fransen M. (2020). Mitochondrial Fission Factor (MFF) Is a Critical Regulator of Peroxisome Maturation. Biochim. Biophys. Acta Mol. Cell Res..

[19] Kustatscher G., Grabowski P., Schrader T.A., Passmore J.B., Schrader M., Rappsilber J. (2019). Co-Regulation Map of the Human Proteome Enables Identification of Protein Functions. Nat. Biotechnol..

[20] Jaipargas E.-A., Mathur N., Bou Daher F., Wasteneys G.O., Mathur J. (2016). High Light Intensity Leads to Increased Peroxule-Mitochondria Interactions in Plants. Front. Cell Dev. Biol..

[21] Dubey G.P., Ben-Yehuda S. (2011). Intercellular Nanotubes Mediate Bacterial Communication. Cell.

[22] Torralba D., Baixauli F., Sánchez-Madrid F. (2016). Mitochondria Know No Boundaries: Mechanisms and Functions of Intercellular Mitochondrial Transfer. Front. Cell Dev. Biol..

[23] Mattes B., Scholpp S. (2018). Emerging Role of Contact-Mediated Cell Communication in Tissue Development and Diseases. Histochem. Cell Biol..

[24] Costello J.L., Passmore J.B., Islinger M., Schrader M. (2018). Multi-Localized Proteins: The Peroxisome-Mitochondria Connection. Proteomics of Peroxisomes.

[25] Wang C., Du W., Su Q.P., Zhu M., Feng P., Li Y., Zhou Y., Mi N., Zhu Y., Jiang D. (2015). Dynamic Tubulation of Mitochondria Drives Mitochondrial Network Formation. Cell Res..

[26] Lavorato M., Iyer V.R., Dewight W., Cupo R.R., Debattisti V., Gomez L., De la Fuente S., Zhao Y.-T., Valdivia H.H., Hajnóczky G. (2017). Increased Mitochondrial Nanotunneling Activity, Induced by Calcium Imbalance, Affects Intermitochondrial Matrix Exchanges. Proc. Natl. Acad. Sci. USA.

[27] Huang X., Sun L., Ji S., Zhao T., Zhang W., Xu J., Zhang J., Wang Y., Wang X., Franzini-Armstrong C. (2013). Kissing and Nanotunneling Mediate Intermitochondrial Communication in the Heart. Proc. Natl. Acad. Sci. USA.

[28] Eisner V., Cupo R.R., Gao E., Csordás G., Slovinsky W.S., Paillard M., Cheng L., Ibetti J., Chen S.R.W., Chuprun J.K. (2017). Mitochondrial Fusion Dynamics Is Robust in the Heart and Depends on Calcium Oscillations and Contractile Activity. Proc. Natl. Acad. Sci. USA.

[29] Vincent A.E., Ng Y.S., White K., Davey T., Mannella C., Falkous G., Feeney C., Schaefer A.M., McFarland R., Gorman G.S. (2016). The Spectrum of Mitochondrial Ultrastructural Defects in Mitochondrial Myopathy. Sci. Rep..

[30] Vincent A.E., White K., Davey T., Philips J., Ogden R.T., Lawless C., Warren C., Hall M.G., Ng Y.S., Falkous G. (2019). Quantitative 3D Mapping of the Human Skeletal Muscle Mitochondrial Network. Cell Rep..

[31] Chung D.J., Madison G.P., Aponte A.M., Singh K., Li Y., Pirooznia M., Bleck C.K.E., Darmani N.A., Balaban R.S. (2022). Metabolic Design in a Mammalian Model of Extreme Metabolism, the North American Least Shrew (*Cryptotis parva*). J. Physiol..

[32] Boardman N.T., Trani G., Scalabrin M., Romanello V., Wüst R.C.I. (2023). Intra-Cellular to Inter-Organ Mitochondrial Communication in Striated Muscle in Health and Disease. Endocr. Rev..

[33] Morcillo P., Cordero H., Ijomone O.M., Ayodele A., Bornhorst J., Gunther L., Macaluso F.P., Bowman A.B., Aschner M. (2021). Defective Mitochondrial Dynamics Underlie Manganese-Induced Neurotoxicity. Mol. Neurobiol..

[34] Quintana-Cabrera R., Scorrano L. (2023). Determinants and Outcomes of Mitochondrial Dynamics. Mol. Cell.

[35] Qin J., Guo Y., Xue B., Shi P., Chen Y., Su Q.P., Hao H., Zhao S., Wu C., Yu L. (2020). ER-Mitochondria Contacts Promote MtDNA Nucleoids Active Transportation via Mitochondrial Dynamic Tubulation. Nat. Commun..

[36] König T., Nolte H., Aaltonen M.J., Tatsuta T., Krols M., Stroh T., Langer T., McBride H.M. (2021). MIROs and DRP1 Drive Mitochondrial-Derived Vesicle Biogenesis and Promote Quality Control. Nat. Cell Biol..

[37] Neuspiel M., Schauss A.C., Braschi E., Zunino R., Rippstein P., Rachubinski R.A., Andrade-Navarro M.A., McBride H.M. (2008). Cargo-Selected Transport from the Mitochondria to Peroxisomes Is Mediated by Vesicular Carriers. Curr. Biol..

[38] Yamashita A., Fujimoto M., Katayama K., Yamaoka S., Tsutsumi N., Arimura S.-I. (2016). Formation of Mitochondrial Outer Membrane Derived Protrusions and Vesicles in *Arabidopsis thaliana*. PLoS ONE.

[39] Zhang L., Trushin S., Christensen T.A., Bachmeier B.V., Gateno B., Schroeder A., Yao J., Itoh K., Sesaki H., Poon W.W. (2016). Altered Brain Energetics Induces Mitochondrial Fission Arrest in Alzheimer’s Disease. Sci. Rep..

[40] Bharti P., Schliebs W., Schievelbusch T., Neuhaus A., David C., Kock K., Herrmann C., Meyer H.E., Wiese S., Warscheid B. (2011). PEX14 Is Required for Microtubule-Based Peroxisome Motility in Human Cells. J. Cell Sci..

[41] Theiss C., Neuhaus A., Schliebs W., Erdmann R. (2012). TubStain: A Universal Peptide-Tool to Label Microtubules. Histochem. Cell Biol..

[42] Barros-Barbosa A., Ferreira M.J., Rodrigues T.A., Pedrosa A.G., Grou C.P., Pinto M.P., Fransen M., Francisco T., Azevedo J.E. (2019). Membrane Topologies of PEX13 and PEX14 Provide New Insights on the Mechanism of Protein Import into Peroxisomes. FEBS J..

[43] Roux A., Cappello G., Cartaud J., Prost J., Goud B., Bassereau P. (2002). A Minimal System Allowing Tubulation with Molecular Motors Pulling on Giant Liposomes. Proc. Natl. Acad. Sci. USA.

[44] Koster G., VanDuijn M., Hofs B., Dogterom M. (2003). Membrane Tube Formation from Giant Vesicles by Dynamic Association of Motor Proteins. Proc. Natl. Acad. Sci. USA.

[45] Waterham H.R., Ferdinandusse S., Wanders R.J.A. (2016). Human Disorders of Peroxisome Metabolism and Biogenesis. Biochim. Biophys. Acta Mol. Cell Res..

[46] Carmichael R.E., Schrader M. (2022). Determinants of Peroxisome Membrane Dynamics. Front. Physiol..

[47] Opaliński Ł., Kiel J.A.K.W., Williams C., Veenhuis M., van der Klei I.J. (2011). Membrane Curvature during Peroxisome Fission Requires Pex11. EMBO J..

[48] Itoyama A., Honsho M., Abe Y., Moser A., Yoshida Y., Fujiki Y., Gould S.J. (2012). Docosahexaenoic Acid Mediates Peroxisomal Elongation, a Prerequisite for Peroxisome Division. J. Cell Sci..

[49] Rodríguez-Serrano M., Romero-Puertas M.C., Sanz-Fernández M., Hu J., Sandalio L.M. (2016). Peroxisomes Extend Peroxules in a Fast Response to Stress via a Reactive Oxygen Species-Mediated Induction of the Peroxin PEX11a. Plant Physiol..

[50] Williams C., Opalinski L., Landgraf C., Costello J., Schrader M., Krikken A.M., Knoops K., Kram A.M., Volkmer R., van der Klei I.J. (2015). The Membrane Remodeling Protein Pex11p Activates the GTPase Dnm1p during Peroxisomal Fission. Proc. Natl. Acad. Sci. USA.

[51] Carmichael R.E., Islinger M., Schrader M. (2022). Fission Impossible (?)—New Insights into Disorders of Peroxisome Dynamics. Cells.

[52] Motley A.M., Hettema E.H. (2007). Yeast Peroxisomes Multiply by Growth and Division. J. Cell Biol..

[53] Nagotu S., Saraya R., Otzen M., Veenhuis M., van der Klei I.J. (2008). Peroxisome Proliferation in Hansenula Polymorpha Requires Dnm1p Which Mediates Fission but Not de Novo Formation. Biochim. Biophys. Acta.

[54] Knoblach B., Rachubinski R.A. (2016). How Peroxisomes Partition between Cells. A Story of Yeast, Mammals and Filamentous Fungi. Curr. Opin. Cell Biol..

[55] Schrader T.A., Carmichael R.E., Islinger M., Costello J.L., Hacker C., Bonekamp N.A., Weishaupt J.H., Andersen P.M., Schrader M. (2022). PEX11β and FIS1 Cooperate in Peroxisome Division Independent of Mitochondrial Fission Factor. J. Cell Sci..

[56] Otera H., Wang C., Cleland M.M., Setoguchi K., Yokota S., Youle R.J., Mihara K. (2010). Mff Is an Essential Factor for Mitochondrial Recruitment of Drp1 during Mitochondrial Fission in Mammalian Cells. J. Cell Biol..

[57] Koch A., Schneider G., Lüers G.H., Schrader M. (2004). Peroxisome Elongation and Constriction but Not Fission Can Occur Independently of Dynamin-like Protein 1. J. Cell Sci..

[58] Costello J.L., Castro I.G., Hacker C., Schrader T.A., Metz J., Zeuschner D., Azadi A.S., Godinho L.F., Costina V., Findeisen P. (2017). ACBD5 and VAPB Mediate Membrane Associations between Peroxisomes and the ER. J. Cell Biol..

[59] Hua R., Cheng D., Coyaud É., Freeman S., Di Pietro E., Wang Y., Vissa A., Yip C.M., Fairn G.D., Braverman N. (2017). VAPs and ACBD5 Tether Peroxisomes to the ER for Peroxisome Maintenance and Lipid Homeostasis. J. Cell Biol..

[60] Kors S., Hacker C., Bolton C., Maier R., Reimann L., Kitchener E.J.A., Warscheid B., Costello J.L., Schrader M. (2022). Regulating Peroxisome-ER Contacts via the ACBD5-VAPB Tether by FFAT Motif Phosphorylation and GSK3β. J. Cell Biol..

[61] Bishop A., Kamoshita M., Passmore J.B., Hacker C., Schrader T.A., Waterham H.R., Costello J.L., Schrader M. (2019). Fluorescent Tools to Analyse Peroxisome-ER Interactions in Mammalian Cells. Contact.

[62] Guillén-Samander A., Leonzino M., Hanna M.G., Tang N., Shen H., De Camilli P. (2021). VPS13D Bridges the ER to Mitochondria and Peroxisomes via Miro. J. Cell Biol..

[63] Baldwin H.A., Wang C., Kanfer G., Shah H.V., Velayos-Baeza A., Dulovic-Mahlow M., Brüggemann N., Anding A., Baehrecke E.H., Maric D. (2021). VPS13D Promotes Peroxisome Biogenesis. J. Cell Biol..

[64] Dziurdzik S.K., Conibear E. (2021). The Vps13 Family of Lipid Transporters and Its Role at Membrane Contact Sites. Int. J. Mol. Sci..

[65] Neuman S.D., Levine T.P., Bashirullah A. (2022). A Novel Superfamily of Bridge-like Lipid Transfer Proteins. Trends Cell Biol..

[66] Ferdinandusse S., Falkenberg K.D., Koster J., Mooyer P.A., Jones R., van Roermund C.W.T., Pizzino A., Schrader M., Wanders R.J.A., Vanderver A. (2017). ACBD5 Deficiency Causes a Defect in Peroxisomal Very Long-Chain Fatty Acid Metabolism. J. Med. Genet..

[67] Yagita Y., Shinohara K., Abe Y., Nakagawa K., Al-Owain M., Alkuraya F.S., Fujiki Y. (2017). Deficiency of a Retinal Dystrophy Protein, Acyl-CoA Binding Domain-Containing 5 (ACBD5), Impairs Peroxisomal β-Oxidation of Very-Long-Chain Fatty Acids. J. Biol. Chem..

[68] Schrader M., Thiemann M., Fahimi H.D. (2003). Peroxisomal Motility and Interaction With Microtubules. Microsc. Res. Tech..

[69] Schrader M., Burkhardt J.K., Baumgart E., Lüers G.H., Spring H., Völkl A., Fahimi H.D. (1996). Interaction of Microtubules with Peroxisomes. Tubular and Spherical Peroxisomes in HepG2 Cells and Their Alteractions Induced by Microtubule-Active Drugs. Eur. J. Cell Biol..

[70] Passmore J.B., Pinho S., Gomez-Lazaro M., Schrader M. (2017). The Respiratory Chain Inhibitor Rotenone Affects Peroxisomal Dynamics via Its Microtubule-Destabilising Activity. Histochem. Cell Biol..

[71] Azadi A.S., Carmichael R.E., Kovacs W.J., Koster J., Kors S., Waterham H.R., Schrader M. (2020). A Functional SMAD2/3 Binding Site in the PEX11β Promoter Identifies a Role for TGFβ in Peroxisome Proliferation in Humans. Front. Cell Dev. Biol..

[72] Sinclair A.M., Trobacher C.P., Mathur N., Greenwood J.S., Mathur J. (2009). Peroxule Extension over ER-Defined Paths Constitutes a Rapid Subcellular Response to Hydroxyl Stress. Plant J..

[73] Barton K.A., Jaipargas E.-A., Griffiths N., Mathur J., Brocard C., Hartig A. (2014). Live Imaging of Peroxisomes and Peroxules in Plants. Molecular Machines Involved in Peroxisome Biogenesis and Maintenance.

[74] Mathur J., Shaikh A., Mathur N. (2018). Peroxisome Mitochondria Inter-Relations in Plants. Proteomics of Peroxisomes.

[75] Barton K., Mathur N., Mathur J. (2013). Simultaneous Live-Imaging of Peroxisomes and the ER in Plant Cells Suggests Contiguity but No Luminal Continuity between the Two Organelles. Front. Physiol..

[76] Purves W.K., Sadava D.E., Orians G.H., Heller H.C., Freeman W.H. (2004). Life: The Science of Biology.

[77] Hamilton N., Kerr M.C., Burrage K., Teasdale R.D. (2007). Analyzing Real-Time Video Microscopy: The Dynamics and Geometry of Vesicles and Tubules in Endocytosis. Current Protocols in Cell Biology.

[78] Schrader M., Wodopia R., Fahimi H.D. (1999). Induction of Tubular Peroxisomes by UV Irradiation and Reactive Oxygen Species in HepG2 Cells. J. Histochem. Cytochem..

[79] Wang B., Van Veldhoven P.P., Brees C., Rubio N., Nordgren M., Apanasets O., Kunze M., Baes M., Agostinis P., Fransen M. (2013). Mitochondria Are Targets for Peroxisome-Derived Oxidative Stress in Cultured Mammalian Cells. Free Radic. Biol. Med..

[80] Mattiazzi Ušaj M., Brložnik M., Kaferle P., Žitnik M., Wolinski H., Leitner F., Kohlwein S.D., Zupan B., Petrovič U. (2015). Genome-Wide Localization Study of Yeast Pex11 Identifies Peroxisome–Mitochondria Interactions through the ERMES Complex. J. Mol. Biol..

[81] Shai N., Yifrach E., van Roermund C.W.T., Cohen N., Bibi C., IJlst L., Cavellini L., Meurisse J., Schuster R., Zada L. (2018). Systematic Mapping of Contact Sites Reveals Tethers and a Function for the Peroxisome-Mitochondria Contact. Nat. Commun..

[82] Binns D., Januszewski T., Chen Y., Hill J., Markin V.S., Zhao Y., Gilpin C., Chapman K.D., Anderson R.G.W., Goodman J.M. (2006). An Intimate Collaboration between Peroxisomes and Lipid Bodies. J. Cell Biol..

[83] Kalutsky M.A., Galimzyanov T.R., Molotkovsky R.J. (2022). A Model of Lipid Monolayer-Bilayer Fusion of Lipid Droplets and Peroxisomes. Membranes.

[84] Thazar-Poulot N., Miquel M., Fobis-Loisy I., Gaude T. (2015). Peroxisome Extensions Deliver the Arabidopsis SDP1 Lipase to Oil Bodies. Proc. Natl. Acad. Sci. USA.

[85] Silva B.S.C., DiGiovanni L., Kumar R., Carmichael R.E., Kim P.K., Schrader M. (2020). Maintaining Social Contacts: The Physiological Relevance of Organelle Interactions. Biochim. Biophys. Acta Mol. Cell Res..

[86] Bonekamp N.A., Sampaio P., de Abreu F.V., Lüers G.H., Schrader M. (2012). Transient Complex Interactions of Mammalian Peroxisomes without Exchange of Matrix or Membrane Marker Proteins. Traffic.

[87] Huybrechts S.J., Van Veldhoven P.P., Brees C., Mannaerts G.P., Los G.V., Fransen M. (2009). Peroxisome Dynamics in Cultured Mammalian Cells. Traffic.

[88] Schrader M., King S.J., Stroh T.A., Schroer T.A. (2000). Real Time Imaging Reveals a Peroxisomal Reticulum in Living Cells. J. Cell Sci..

[89] Chornyi S., IJlst L., van Roermund C.W.T., Wanders R.J.A., Waterham H.R. (2020). Peroxisomal Metabolite and Cofactor Transport in Humans. Front. Cell Dev. Biol..

[90] Klar T.A., Jakobs S., Dyba M., Egner A., Hell S.W. (2000). Fluorescence Microscopy with Diffraction Resolution Barrier Broken by Stimulated Emission. Proc. Natl. Acad. Sci. USA.

[91] Rust M.J., Bates M., Zhuang X. (2006). Sub-Diffraction-Limit Imaging by Stochastic Optical Reconstruction Microscopy (STORM). Nat. Methods.

[92] Betzig E., Patterson G.H., Sougrat R., Lindwasser O.W., Olenych S., Bonifacino J.S., Davidson M.W., Lippincott-Schwartz J., Hess H.F. (2006). Imaging Intracellular Fluorescent Proteins at Nanometer Resolution. Science.

[93] Heilemann M., van de Linde S., Schüttpelz M., Kasper R., Seefeldt B., Mukherjee A., Tinnefeld P., Sauer M. (2008). Subdiffraction-Resolution Fluorescence Imaging with Conventional Fluorescent Probes. Angew. Chem. Int. Ed. Engl..

[94] Van de Linde S., Löschberger A., Klein T., Heidbreder M., Wolter S., Heilemann M., Sauer M. (2011). Direct Stochastic Optical Reconstruction Microscopy with Standard Fluorescent Probes. Nat. Protoc..

[95] Legant W.R., Shao L., Grimm J.B., Brown T.A., Milkie D.E., Avants B.B., Lavis L.D., Betzig E. (2016). High-Density Three-Dimensional Localization Microscopy across Large Volumes. Nat. Methods.

[96] Spahn C.K., Glaesmann M., Grimm J.B., Ayala A.X., Lavis L.D., Heilemann M. (2018). A Toolbox for Multiplexed Super-Resolution Imaging of the E. Coli Nucleoid and Membrane Using Novel PAINT Labels. Sci. Rep..

[97] Hoboth P., Šebesta O., Sztacho M., Castano E., Hozák P. (2021). Dual-Color DSTORM Imaging and ThunderSTORM Image Reconstruction and Analysis to Study the Spatial Organization of the Nuclear Phosphatidylinositol Phosphates. MethodsX.

[98] Hoboth P., Šebesta O., Hozák P. (2021). How Single-Molecule Localization Microscopy Expanded Our Mechanistic Understanding of RNA Polymerase II Transcription. Int. J. Mol. Sci..

